# Liver Involvement in Acute Respiratory Infections in Children and Adolescents – Results of a Non-interventional Study

**DOI:** 10.3389/fped.2022.840008

**Published:** 2022-03-29

**Authors:** Wolfgang Kamin, Ortwin Adams, Peter Kardos, Heinrich Matthys, Norbert Meister, Christian P. Strassburg

**Affiliations:** ^1^Children’s Hospital, Evangelic Hospital, Hamm, Germany; ^2^Faculty of Medicine, Pomeranian Medical University, Szczecin, Poland; ^3^Institute for Virology, Heinrich-Heine University, Düsseldorf, Germany; ^4^Group Practice and Centre for Pneumology, Allergy and Sleep Medicine at Red Cross Maingau Hospital, Frankfurt am Main, Germany; ^5^Department of Pneumology, University Hospital, Freiburg, Germany; ^6^Paediatric Practice, Bindlach, Germany; ^7^Department of Internal Medicine I, University Hospital, Bonn, Germany

**Keywords:** acute respiratory tract infections, children, adolescents, liver involvement, non-interventional study

## Abstract

**Background:**

In children and adults with acute respiratory tract infections (ARTI), elevations of serum liver enzyme activities are frequently observed in clinical practice. However, epidemiological data particularly in the pediatric population are very limited. The aim of this study was to assess the incidence of hepatic involvement, to identify the viruses and to analyze risk factors in children and adolescents with ARTI in a real-world setting.

**Methods:**

We report on a prospective, multicenter, non-interventional study with 1,010 consecutive patients aged 1–17 years with ARTI who consulted a physician within 5 days after onset of symptoms. Laboratory blood tests and PCR virus detection in nasopharyngeal lavage were performed at first presentation and after 3–7 days. Patients with elevated activities of serum liver enzymes (ASAT, ALAT, and γ-GT) were determined in local laboratories and values were normalized by dividing by the individual upper limit of the normal range (ULN). The resulting index (<1 means below ULN, >1 means above ULN) allowed to compare results from laboratories with different reference ranges.

**Results:**

Laboratory test results of 987 patients were available at first visit. 11.1% (95% CI: 9.2–13.3%) exhibited an elevation of ASAT, ALAT, and/or γ-GT activities. Virus DNA or RNA was identified in nasopharyngeal lavages of 63% of the patients. 12.2% of patients with positive PCR and 9.7% of those with negative PCR (*p* = 0.25) had elevated serum liver enzyme activities. The highest rates were observed in patients with a positive result for influenza B virus (24.4%) followed by human metapneumovirus (14.6%), and human coronavirus (others than SARS-CoV-2) (13.6%). The rate of children and adolescents with ARTI and elevation of serum liver enzyme activities correlated with the virus species and with overweight of the patients but did not differ in patients with or without previous medication intake.

**Conclusion:**

Elevated enzyme activities are present in about 10% of children and adolescents with ARTI. In our cohort, these elevations were mild to moderate; probably resulting from an inflammation process with hepatic involvement.

## Introduction

Up to 90% of acute respiratory tract infections (ARTI) are of viral etiology mostly caused by rhinoviruses, respiratory syncytial virus (RSV), human metapneumovirus (hMPV), influenza virus, human coronavirus (HCoV), parainfluenza viruses and adenoviruses (1–3). Particularly children commonly encounter infections with multiple virus species (2). The respiratory tract as the first entry point is initially affected when the viral replication causes destruction of the airway tissue by cell loss, goblet cell hyperplasia, altered mucus secretion, and/or biochemistry. Consequently, sore throat, headache, sneezing, runny nose and nasal congestion as clinical signs usually appear early in the course of the disease subsequently followed by cough. During the viremic phase characterized by fever and chills, the virus may spread to other tissues and organs including the liver. Observations in routine clinical practice indicate that pediatric patients with ARTI exhibit elevated serum liver enzyme activities (ELEA) as an indicator of hepatic involvement.

A retrospective study reported mild elevation of transaminase activities and hepatic inflammation in more than half of the pediatric patients suffering from lower respiratory tract infections caused by hepatotropic adenoviruses (4). According to a systematic review on RSV infections, the virus or its genetic material could be isolated from cerebrospinal fluid, peripheral blood, myocardium, and the liver indicating a systemic dissemination (5). Several studies indicate an involvement of the liver in influenza infections (6–8). Out of 559 children presenting with abnormal liver enzyme activities in a Korean hospital, 323 cases (57.8%) were attributable to an infection (9). Among these, viral respiratory tract infection was the largest subgroup (111/559 children, 19.8%) (10).

Although published evidence indicates that hepatic inflammation in patients suffering from ARTIs may not be an exception, the pathogenesis of liver involvement is not well understood. The role of potentially contributing factors such as drug intake or overweight has not been investigated systematically. Prospective studies elucidating the prevalence of and risk factors for hepatic involvement in patients suffering from ARTIs are therefore needed.

We report on a multicenter, non-interventional study performed in children and adolescents who suffered from an uncomplicated lower or upper ARTI, for which they received an individually chosen treatment or no treatment. The objectives of the study were to assess the incidence of hepatic involvement, to identify the viruses and to analyze risk factors. It was conducted in a practice setting under real-world conditions, which allowed to include a significant number of patients.

## Patients and Methods

### Study Design and Schedule

The prospective, multi-center, non-interventional study included consecutive patients meeting the selection criteria. They underwent a study entry examination (visit 1) with documentation of the patient’s history including previous medication intake, physical examination, blood sampling, and nasopharyngeal lavage. The study exit examination (visit 2) took place 3–7 days later, during which laboratory tests were repeated. The therapy was up to the decision of the attending physicians as the study did not investigate a treatment modality.

Up to 3 ml of blood were taken for laboratory investigations which were performed in local laboratories. Aspartate aminotransferase (ASAT), alanine aminotransferase (ALAT), gamma glutamyltransferase (γ-GT), alkaline phosphatase (AP), bilirubin (total, direct, and indirect), total protein and C-reactive protein (CRP) were determined for all patients. For in-patients, a full blood count, serum electrolytes, a venous blood gas analysis, as well as an analysis of the oxygen saturation of the blood using a pulse oximeter was carried out additionally.

For the detection of virus DNA or RNA, a nasopharyngeal lavage with 3 ml of physiological saline solution was performed. Samples were analyzed by multiplex polymerase chain reaction (PCR) (10) covering influenza A (including H1N1sw) and B virus, parainfluenza 1, 2, and 3 virus, RSV (A and B), hMPV (A and B), HCoV (other than SARS-CoV-2) strains 229E, OC43 and NL63, rhino-, entero-, adeno-, and bocaviruses.

### Participants and Selection Criteria

Male and female in- and out-patients from 1 to 17 years of age, who suffered from an upper or lower ARTI, e.g., the common cold, rhinitis, sinusitis, pharyngitis, bronchitis, interstitial pneumonia were eligible for participation. We excluded patients with severe infections requiring mechanical ventilation or circulatory support, patients with congenital defects or metabolic disturbances potentially interfering with the interpretation of their hepatic laboratory parameters, patients with bronchopneumonia, pleuropneumonia, or lobar pneumonia, as well as patients with pre-existing jaundice or an infection already persisting for more than 5 days.

### Outcomes Criteria

The elevated serum liver enzyme (ASAT, ALAT, and γ-GT) activities were measured in local laboratories, each using different normal ranges which also depended on gender and age of the patients. These values were normalized by dividing by the upper limit of the normal range (ULN) to yield comparable values across laboratories. The resulting indices allow a common interpretation: values < 1 indicate original values below the ULN, values above > 1 indicate elevations. An index value of 2, for example, thus corresponds to a measured value that is twice as high as the ULN. Elevation of serum liver enzyme activity (ELEA) was defined as an index value >1 at least for one of ASAT, ALAT, or γ-GT.

Children were defined to be of normal weight with a body mass index (BMI) at or below the 90th percentile of their age group, overweight with BMI between above the 90th and at or below the 97th percentile, and obese with BMI above the 97th percentile (11).

At study entry visit, we asked for previous medication use during the last 30 days until the time point of the laboratory test of the first visit. This was independent of the fact whether their intake was continued during the study period. In cases where previous medication intake remained unclear due to incomplete or inconsistent data, this was assessed as no previous medication intake.

### Statistical Methods and Sample Size

Explorative statistical analyses were pre-defined in a statistical analysis plan. They investigate the incidence of elevated transaminases in the total patient sample as well as in subsets defined by demographic characteristics, previous medication, and virus detection.

Sample size calculation based on the assumption that the ELEA incidence is 5–8% and the virus detection rate is between 20 and 25%. Consequently, 1,000 children and adolescents were planned to be included for obtaining a 95% confidence interval for the incidence of ELEA that excluded the value of zero in the subgroup of children with a positive virus test. All confidence intervals (CIs) for proportions are exact intervals calculated according to Pearson and Clopper (12).

## Results

### Study Participants

**FIGURE 1:**
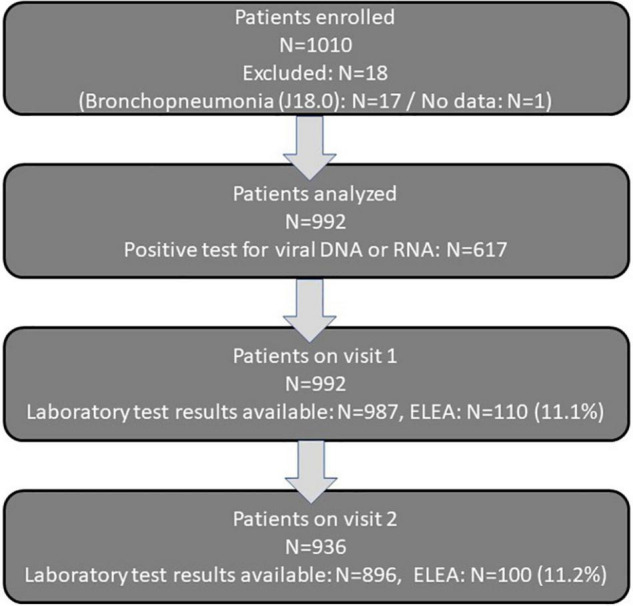
Patient flow in the uncontrolled observational study.

Between January 2014 and December 2016, a total of 1,010 patients were included in six pediatric practices (609 out-patients) and one pediatric hospital (401 patients) in Germany. Seventeen patients with bronchopneumonia and one patient with missing data (results not available at data review) were excluded from the analysis. The resulting population consisted of 992 patients with valid data. For 987 of them laboratory test results were available from visit 1, 896 from visit 2 and for 892 from both visits (measures available for four patients from visit 2 but not from visit 1) (Figure 1).

The most frequent main diagnoses at study entry were unspecified acute upper respiratory tract infection in about half of the participants, followed by acute unspecified bronchitis, and unspecified acute lower respiratory infection. Main demographic, anthropometric and anamnestic characteristics of the study participants are shown in Table 1.

**TABLE 1:** Patient demographics and baseline characteristics.

	Number (*n*, %)
**Sex, *n* = 992**	
Male	572 (57.7%)
Female	420 (42.3%)
Age (years), *n* = 992	6.2 ± 4.6
**Age group, *n* = 992**	
1–5 years	576 (58.1%)
6–12 years	307 (31.0%)
>12 years	109 (11.0%)
Body Mass Index, *n* = 937	17.5 ± 3.7
**Main diagnosis at inclusion (acc. to ICD-10), *n* = 991**	
J06.9 – Acute upper respiratory infection, unspecified	478 (48.2%)
J20.9 – Acute bronchitis, unspecified	208 (21.0%)
J22 – Unspecified acute lower respiratory infection	54 (5.5%)
J11.1 – Influenza with other respiratory manifestations, virus not identified	48 (4.8%)
J02.9 – Acute pharyngitis, unspecified	38 (3.8%)
J05.0 – Acute obstructive laryngitis [croup]	27 (2.7%)
J18.9 – Pneumonia, unspecified	25 (2.5%)
Other disorders of the respiratory system	113 (11.5%)

### Incidence of Elevated Liver Enzyme Activities

For 892 individuals, liver enzyme activities were available for both visits. Sixty-three of them (7.1%, CI: 5.5–9%) had elevated enzyme activities at first visit and second visit, 41 (4.6%) had elevations only at the first and 37 (4.1%) only at the second visit. Thus, in total 141 patients (15.8%) had at least one elevated activity during at least one visit. Table 2 presents the number and percentage of children with elevated ALAT, ASAT, and γ-GT activity at visit 1, 2, or both.

**TABLE 2:** Numbers and percentage of patients with elevated serum liver enzyme activities (ELEA) at visits 1 (V1) and 2 (V2).

Laboratory parameter	Patients (N) with valid data for both visits	Value elevated at V1 and normal at V2	Value elevated at V1 and elevated at V2	Normal value at V1 and elevated value at V2
ALAT	886	11 (1.2%)	18 (2.0%)	17 (1.9%)
ASAT	886	37 (4.2%)	24 (2.7%)	22 (2.5%)
γ-GT	862	8 (0.9%)	29 (3.4%)	20 (2.3%)

At first visit, an elevated activity of at least one liver enzyme was present in 110 (including additional 6 patients for whom only data at visit 1 were available) out of 987 patients (11.1%, 95% CI: 9.2–13.3%). 89 patients showed an elevated activity of one enzyme, 16 had elevations of two, and 5 of all three enzymes. In 93 children, elevations were below the index value 2 (measured value/ULN), in 10 the index value was ≥2 and <3 and in 6 it was ≥3.

At the second visit, elevated activities were present in 100/896 patients (11.2%, 95% CI: 9.2–13.4%; for four patients only data for visit 2 were available). Elevations were below the index value 2 (measured value/ULN) in 79 children, ≥2 and <3 in 14 children, and ≥3 in 7 children.

In 13 patients, pronounced elevations were present with index values ≥3 (both visits). A marked peak elevation (12-times ULN) was only seen once in one patient. Five patients showed a mild hyperbilirubinemia with an index value ≥2 for direct bilirubin; none of them had marked elevations of transaminases or γ-GT (ULN < 2). When applying the criteria of the US Food and Drug Administration (FDA), i.e., transaminases ≥3× ULN and total bilirubin index value ≥2× ULN, no indication for hepatocellular injury was seen in any patient (13).

### Relationship Between Elevated Liver Enzyme Activities and Virus Detection by Polymerase Chain Reaction

Virus DNA or RNA was identified in 617 patients (63%). In 529 of these (86.2%), DNA or RNA of only one species was detected. In the others, up to four different virus species were seen. Detection rates were higher in children aged 1–5 years (73.8% of *n* = 569) than in those 6–12 years of age (49.3% of *n* = 304) or 13–17 years (43.9% of *n* = 107). Detection rates were also higher in in-patient (71.7% of *n* = 368) than in out-patient settings (57.7% of *n* = 612).

At visit 1, laboratory test results as well as a valid PCR result were available in 976 patients. ELEA were observed in 75/614 patients (12.2%; 95% CI: 9.7–15.1%) with a positive PCR test and in 35/362 patients (9.7%; 95% CI: 6.8–13.2%) with a negative PCR test.

Rhinoviruses, RSV (A or B) and influenza B were the 3 most frequently detected virus species in this study. The highest number of transaminase activity elevations was detected in patients positive for influenza B virus, followed by hMPV (A or B), HCoV, RSV (A or B), influenza A, and parainfluenza virus (Table 3).

**TABLE 3:** Viruses detected in the whole sample and proportion of patients with elevated serum enzyme activities (ELEA) at visit 1.

Virus species	Number of Patients	Patients with ELEA^#^
Any detected virus	614*	75 [12.2% (9.7%, 15.1%)]
Influenza B	90	22 [24.4% (16.0%, 34.6%)]
Human metapneumovirus (A, B)	41	6 [14.6% (5.6%, 29.2%)]
Human coronavirus (229E, OC43, NL63)	59	8 [13.6% (6.0%, 25.0%)]
Respiratory syncytial virus (A, B)	105	14 [13.3% (7.5%, 21.4%)]
Influenza A (H1N1, H3N2)	39	5 [12.8% (4.3%, 27.4%)]
Parainfluenza (1, 2, 3)	69	8 [11.6% (5.1%, 21.6%)]
Enterovirus	26	2 [7.7% (0.9%, 25.1%)]
Rhinovirus	178	12 [6.7% (3.5%, 11.5%)]
Adenovirus	62	4 [6.5% (1.8%, 15.7%)]
Human bocavirus	42	2 [4.8% (0.6%, 16.2%)]

*For three patients no liver enzyme activities were determined. ^#^Percent values based on no. of patients with confirmed viral nucleic acid.

**FIGURE 2:**
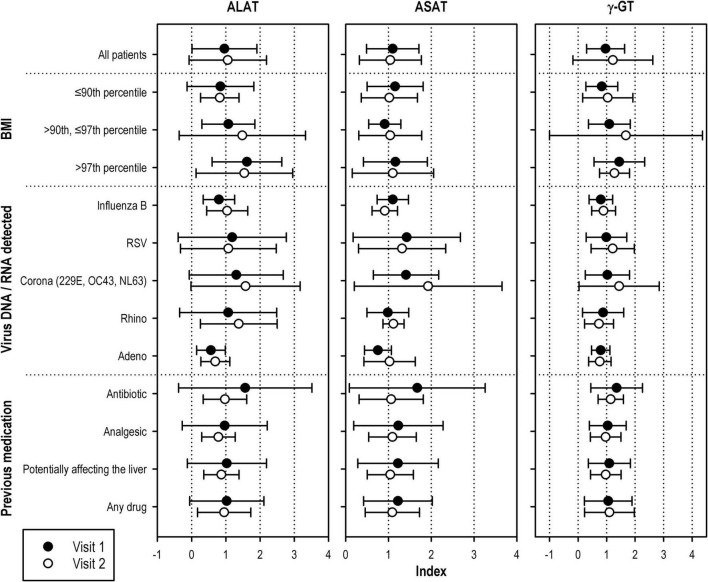
ALAT index ASAT index, and γ -GT index of patients with at least one elevated serum liver enzyme activity at visit 1 or visit 2 (means ± standard deviations). Only subgroups with at least five patients at visit 2 are displayed. Laboratory values were normalized by dividing by the upper limit of the normal range (ULN). The resulting index (<1, below ULN;>1, above ULN) allowed to compare results from laboratories with different reference ranges.

Influenza virus A and B, RSV (A or B), hMPV (A or B), and HCoV were associated with an at least 2% increase in the proportion of patients with ELEA, up to a 2.5-fold increase in case of influenza B virus (Figure 2). By comparison, patients affected by rhino-, entero-, and adenoviruses or by human bocavirus showed lower activity elevation rates than patients in whom no virus could be detected.

### Relationship Between Elevated Liver Enzyme Activities and Previous Medication

Out of 992 study participants, 373 (37.6%) had at least one previous medication during the last 30 days prior to study inclusion. Most frequent were analgesic or antiphlogistic drugs [169 patients, 17.0% of 992, notably ibuprofen (134 patients, 13.5%) and paracetamol (55 patients, 5.5%)] followed by mucolytics (75 patients, 7.6%), antibiotics (64 patients, 6.5%), β-2- (47 patients, 4.7%) or α-1-sympathomimetic drugs (41 patients, 4.1%). All other types of drugs were used by less than 4% of participants.

At visit 1, the proportion of patients with ELEA was 12.9% (48/372 patients with elevated liver enzymes; 95% CI: 9.7–16.7%) in the group with previous medication and 10.1% (62/615 patients; 95% CI: 7.8–12.7%) without previous medication.

Elevated liver enzyme activities rates in patients taking drugs adversely affecting the liver (according to the patient information leaflet) were in the same range than the whole group. A slightly higher number of elevated aminotransferase activities was observed in patients taking analgesics or antibiotics (Figure 2).

### Relationship Between Elevated Liver Enzyme Activities and Body Mass Index

Body mass index (BMI) of the participants increased with age, from a median (range) of 16.0 (11.4–28.5) in children 1–5 years old to 21.8 (15.2–45.3) in adolescents ≥13 years of age. Overweight [23/92, 25.0% (16.6%, 35.1%)] or obese [9/44, 20.5% (9.8%, 35.3%)] patients were at higher risk of enzyme activity elevation than those with normal weight [73/796, 9.2% (95% CI: 7.3–11.4%)]. While this applied to all age groups, overweight or obese adolescents >12 years of age were at a substantially higher risk of ELEA than younger children.

Figure 2 shows means and standard deviations of index values (measured value/ULN) for ASAT, ALAT, and γ-GT of patients with ELEA at visit 1. The figure shows that, among all subsets of patients, adolescents ≥13 years of age as well as patients with a BMI above the 90th percentile of this age group and, even more so, those above the 97th percentile presented with the highest indices for ALAT and γ-GT.

### Relationship Between Elevated Liver Enzyme Activities and Study Site

Among the 6 participating sites, ELEA ranged between 7.9% (20/254 patients) and 18.8% (25/133 patients) at visit 1. Two of the three centers with the highest ELEA rates had proportions of patients affected by influenza B or RSV viruses that were clearly above the study average, which is the likely explanation for this finding. The two largest centers (one of which was the participating pediatric hospital), which contributed 639 out of 987 patients (64.7%), reported the lowest rates of elevated transferase activities.

## Discussion

The American Gastroenterological Association (AGA) reported that about 1–4% of the asymptomatic population may have elevated serum liver chemistry parameters (14). Although differences between estimates may arise from different definitions of “elevation,” it is not surprising that mild elevations of liver enzyme activities are a common incidental finding in asymptomatic patients in primary care (15). According to the National Health and Nutrition Examination Survey conducted in the United States around the turn of the millennium, the prevalence of elevated ALAT, ASAT, or either ALAT or ASAT were 7.3, 3.6, and 8.1%, respectively, after excluding participants who tested positive for hepatitis C virus (HCV) antibody or reported excessive alcohol consumption (16). While this prevalence applies to the general population, epidemiological data in children and adolescents are largely missing.

A major strength of the study was the large cohort of 1,010 children and adolescents with acute respiratory infections, which was systematically investigated for liver enzyme activities. To our knowledge, this represents the first epidemiological study to this end. In the studied cohort, the prevalence of ELEA was 11.1% (95% CI: 9.2–13.3%) at visit 1 and 11.2% (95% CI: 9.2–13.4%) at visit 2. The elevations detected in the study were mostly mild, probably reflecting a generalized inflammatory process impacting various tissue and organs including the liver. Moderate and partly persisting ELEA of ULN ≥ 3 were only seen in 13 children. Although the liver has a considerable regenerative capacity, injury to hepatocytes sufficient to cause mild hyperbilirubinemia (i.e., a bilirubin ULN > 2) already represents a significant liver injury in some patients. Mild increase of direct bilirubin in one patient without pronounced ELEA and ELEA with index value ≥3 in 13 patients without hyperbilirubinemia do not indicate hepatocellular damage in our cohort (13). Hepatic work-up with thorough liver-specific diagnostics were therefore not carried out. Consequently, an evaluation of the clinical condition regarding liver involvement has not been performed and represents a subject for further research. This is justified based on data from a study on idiopathic asymptomatic aminotransferase elevation in healthy children where ALAT levels ranged from 1.5 to 15.9-fold of the ULN. Although it resolved spontaneously in most children, the abnormality persisted for a median of 10 months. Comprehensive workup found the etiology only in half of the patients, and the most common diagnoses were fatty liver and CMV hepatitis (17).

An elevation of ASAT or ALAT activities for various causes is rather common also in the pediatric population (9, 15, 18–20). Out of 559 children with abnormal liver function presenting to a hospital in Korea, 19.8% patients had a viral infection in the respiratory tract (18). Our study provides numerical evidence for the large and clinically important yet often medically underserved subpopulation of children and adolescents suffering from ARTI.

Viral DNA or RNA was detected in 63% of patients in our cohort. This rate largely depends on the test system used since only nucleic acids can be detected for which the virus specific primers are applied. The rate of ELEA in patients with positive PCR was only slightly higher than in patients with negative test results. The highest risk was observed for patients with influenza B infection (2.5-fold increase over patients with a negative assay), followed by hMPV and RSV (about 1.5-fold increase). For influenza B infections, elevated serum liver enzyme activities have been found in both animal models and in humans (21–23). HCoV (other than SARS-CoV-2) was not associated with notable increase in ELEA. In a cohort of COVID-19 patients, the levels of ALAT/ASAT elevation detected in the study were mild and more commonly associated with higher inflammatory indices, such as elevated C-reactive protein (CRP) and procalcitonin (PCT). Although this may suggest a role for uncontrolled inflammation in the pathogenesis of COVID-19 related liver injury, this observation cannot justify the presence of abnormal liver function tests found in patients with mild SARS-CoV-2 infection (24).

Pre-treated study participants had only slightly higher enzyme activities than those without previous medication. Patients who took antibiotics before showed an about 0.5-fold increase compared to untreated individuals. This could, however, also be attributable to the fact that patients with more severe infections may have been more likely to receive antibiotics, analgetic and antiphlogistic substances. Interestingly, transaminase activities in patients pre-treated with drugs with known adverse effects on the liver were in the range of those with any previous medication.

We found that overweight and obese study participants were at a notably higher risk of elevated liver enzyme activities compared to those with normal or low BMI. Of note, the definitions for overweight (BMI above the 90th percentile) and for adipositas (BMI above the 97th percentile) in our study derived from the German KiGGS study (11) and are slightly higher than the ones applied internationally (overweight above the 85th percentile, adipositas above the 95th percentile) (25). The subset difference was particularly pronounced in adolescents where the variability of BMI was larger than in younger patients and where a higher proportion of participants were obese. These findings are consistent with previous publications stating that obesity is the most frequent cause of hypertransaminasemia in both adults and children (20). In a cross-sectional study, prepubertal children with obesity were shown to have higher values for transaminase activities than children with normal weight (26). In another study with 234 obese children aged 6 years and older, 40% even suffered from Non-alcoholic Fatty Liver Disease (27). Guidelines recommend elevated ALAT alone or in combination with ultrasound as diagnostic screening methods. In our study, individuals with BMI above the 97th percentile presented with the highest indices for ALAT and γ-GT. The correlation might therefore exist between ELEA and obesity in addition to the respiratory tract infection.

General limitations of uncontrolled observational research may also apply to this study, e.g., some inconsistent or missing data, e.g., concerning previous medication, which could not be clarified. Moreover, as our study setting did not include a control group, we obtained an estimate for the incidence of ELEA in pediatric patients with ARTI and in several clinically important subsets but could not compare these results to those of a healthy control group. However, clinical practice also benefits from pragmatically designed studies reflecting real-life practice in a relatively large cohort with only very few exclusion criteria. Controlled clinical trials in general seek for standardized variables to optimize internal validity (28). This could be the subject of further studies.

## Conclusion

In a pediatric cohort seeking medical care for ARTI under real-world conditions, we found a relevant rate of elevations of serum liver enzyme activities. The incidence was higher in the presence of come viruses and in patients with overweight or obesity. More rigorous research may elucidate if a causal relationship is present. In our sample, elevations were mostly mild to moderate. However, special attention should be given to children with severe clinical courses especially when they are obese.

## Data Availability Statement

The datasets presented in this article are not readily available because raw data cannot be shared both due to ethical reasons and to data protection laws. To the extent permitted by law, the data required for validation purposes have already been disclosed in results reports on corresponding data bases. All relevant data are within the article. Requests to access the datasets should be directed to the corresponding author (WK), paediatrie.hamm@valeo-kliniken.de.

## Ethics Statement

This study involving human participants was reviewed and approved by the Ethics Committee of Ärztekammer Westfalen-Lippe and Medical Faculty of the University of Münster. Written informed consent to participate in this study was provided by the participants’ legal guardian/next of kin. The study was conducted in Germany in accordance with the Declaration of Helsinki (version 2008) and recorded in the German Clinical Trials Register (2014/01/09, DRKS-ID: DRKS00005592).

## Author Contributions

WK: study concept and design. WK, OA, PK, HM, NM, and CS: substantial contributions to the conception of the work, interpretation, and discussion of the data. WK and NM: recruiting children and adolescents and sampling. OA: virus detection. All authors read, revised, and edited the drafted manuscript, and approved the final version to be published.

## Conflict of Interest

WK, OA, PK, HM, NM, and CS received honoraria from Dr. Willmar Schwabe GmbH & Co. KG, Karlsruhe, Germany. The authors declare that this study received funding from Dr. Willmar Schwabe GmbH & Co. KG, Karlsruhe, Germany. The funder was involved in the study design, analysis, and preparation of the manuscript. Editorial/medical writing support was provided by Andreas Völp, Psy Consult Scientific Services, Hamburg, Germany and was funded by Dr. Willmar Schwabe GmbH & Co. KG. Final decision to submit the manuscript for publication remained with the authors.

## Publisher’s Note

All claims expressed in this article are solely those of the authors and do not necessarily represent those of their affiliated organizations, or those of the publisher, the editors and the reviewers. Any product that may be evaluated in this article, or claim that may be made by its manufacturer, is not guaranteed or endorsed by the publisher.

## Abbreviations

ALAT: alanine aminotransferase
AP: alkaline phosphatase
ARTI: acute respiratory tract infection
ASAT: aspartate aminotransferase
BMI: body mass index
CI: confidence interval
CRP: C-reactive protein
ELEA: elevated liver enzyme activities
γ -GT: gamma-glutamyltransferase
HCoV: human coronavirus
hMPV: human metapneumovirus
PCR: polymerase chain reaction
RSV: respiratory syncytial virus
ULN: upper limit of the normal range.
